# Regulatory B cells: heterogeneity, immunosuppressive networks, and contributions to autoimmune pathogenesis

**DOI:** 10.3389/fimmu.2026.1808399

**Published:** 2026-04-10

**Authors:** Yikai Liu, Xiaochao Zhang, Qun Ji, Yuhai Zhang, Weiping Wei, Zewei Mo, Te Chen

**Affiliations:** 1 Department of Endocrinology, Hainan General Hospital, Hainan Affiliated Hospital of Hainan Medical University, Haikou, Hainan, China; 2 Department of Dermatology, Hainan General Hospital, Hainan Affiliated Hospital of Hainan Medical University, Haikou, Hainan, China; 3 Division of Joint Surgery, Department of Orthopaedics, Hainan General Hospital, Hainan Affiliated Hospital of Hainan Medical University, Haikou, Hainan, China

**Keywords:** autoimmune diseases, heterogeneity, IL-10, immune regulation, regulatory B cells

## Abstract

B cells contribute to humoral immunity by producing antibodies, presenting antigens, and secreting cytokines. Traditionally, B cells have been viewed as predominantly pro-immunogenic effectors that promote adaptive immune responses. However, accumulating evidence has identified a distinct B cell subset with immunosuppressive properties, termed regulatory B cells (Bregs), which restrain pathogenic T-cell responses by secreting anti-inflammatory cytokines including interleukin-10 (IL-10), IL-35, and transforming growth factor β (TGF-β), and by expressing immunoregulatory surface molecules such as T cell immunoglobulin and mucin domain 1 (Tim-1), programmed cell death protein 1 (PD-1), and Fas ligand (FasL). Through these mechanisms, Bregs contribute to immune homeostasis and the maintenance of immune tolerance. Moreover, published studies have linked Bregs to the progression of autoimmune diseases, and substantial experimental data support quantitative and functional abnormalities of Bregs across multiple autoimmune conditions. In this review, we summarize current knowledge regarding the developmental origin, phenotypic features, and biological functions of Bregs, with emphasis on their roles in autoimmune diseases. A deeper understanding of Bregs in autoimmunity may provide a promising foundation for future immunotherapeutic strategies.

## 1 Introduction

The immune system protects the host from invading pathogens and other non-self components through coordinated innate and adaptive immune responses (1). In most cases, these responses are accompanied by inflammation, which is essential for pathogen clearance and tissue defense (2). However, when immune activation is excessive, prolonged, or improperly controlled, it can damage host tissues and contribute to the development of immune-mediated disorders, including autoimmune diseases. Under physiological conditions, immune homeostasis is maintained by complex regulatory networks involving multiple immune cell populations and soluble mediators that collectively restrain the magnitude and duration of immune responses. Disruption of this regulatory balance is a major driver of autoimmune initiation and progression (3).

With advances in immunology, a suppressive B cell subpopulation, collectively termed regulatory B cells (Bregs), has been increasingly recognized (4). Bregs exert immunomodulatory effects through the secretion of anti-inflammatory cytokines and the expression of inhibitory surface molecules, thereby playing an essential role in maintaining immune tolerance (5). Accumulating evidence indicates that abnormalities in Breg number, phenotype, and function are closely associated with the onset and progression of autoimmune diseases (6). However, major questions remain unresolved regarding their phenotypic heterogeneity, developmental origin, context-dependent regulatory programs, and disease-specific pathogenic relevance. A clearer understanding of these issues is essential for the development of more precise immunoregulatory therapies. In this review, we summarize current knowledge of the developmental origin, phenotypic characteristics, and biological functions of Bregs, with particular emphasis on their heterogeneity, immunosuppressive networks, and contributions to autoimmune pathogenesis.

## 2 Origin and phenotype of Bregs

### 2.1 Development of Breg research

Research on Bregs initially emerged from animal studies. As early as 1974, several investigators reported that adoptive transfer of B cell–depleted splenocytes into guinea pigs failed to suppress delayed-type hypersensitivity, suggesting that B cells can exert negative immune regulatory effects, at least in part by suppressing T-cell function (7, 8). In 1990, Wolf et al (9). found in a mouse model of experimental autoimmune encephalomyelitis (EAE) that B cells were involved in suppressing the T cell-mediated autoimmune response and that B cell-deficient mice rarely recovered after induction of EAE. In a published study, Mizoguchi et al (10) demonstrated that B cell–deficient mice with experimental inflammatory bowel disease (IBD) developed more severe colitis, further implying the existence of suppressive B cell subsets in immune-mediated disease. They later coined the term “regulatory B cells,” describing a subset that negatively regulates immune inflammation primarily through IL-10 production. These cells are induced during inflammation and can downregulate inflammatory cascades mediated by IL-1 upregulation and STAT3 activation (11). Furthermore, Fillatreau et al (12). clarified that immunosuppressive activity is not a universal property of all B cells but is enriched in subsets capable of producing IL-10. In IL-10–deficient settings, EAE could not be effectively ameliorated and pathogenic Th1 responses were enhanced. Subsequent studies expanded this concept by demonstrating that B cells can mediate immunosuppression through secretion of multiple inhibitory cytokines, including IL-10, IL-35 and transforming growth factor β (TGF-β) (13).

### 2.2 Phenotypic characterization of Bregs

To date, no unique surface markers or definitive lineage-specific transcription factors have been identified for Bregs (6, 14) As a result, Bregs are generally defined functionally as B cell subsets that suppress inflammatory responses rather than promote them. Numerous studies have described phenotypically distinct Breg subsets in mice and humans, each contributing to immune homeostasis. The reasons for the overlap among reported Breg phenotypes remain unclear, but it is plausible that different disease contexts, stimuli, and immune microenvironments elicit distinct regulatory b cell states (15).

In murine studies, several groups have identified transitional 2 marginal-zone precursor (T2-MZP) cells as a Breg-enriched population. Predominantly found in the spleen of naïve mice, T2-MZP B cells can inhibit T-cell activation and Th1 responses, thereby reducing inflammation and promoting allograft tolerance (16, 17). In addition, Tim^+^ B cells (18), MZB cells (19), B-1a cells (20) and regulatory plasma cells (21) have also been described as Breg subsets. Beyond classical subsets, B cell–derived cytokines may themselves define regulatory programs. For example, IL-35 secreted by B cells can suppress inflammatory bowel disease, and these cells are termed IL-35–producing B cells (22). Studies in experimental autoimmune disease models have shown that mice with a selective lack of TGF-β in B cells (B-TGF-β1-/-) develop an exacerbated form of EAE compared to wild-type controls. This suggests that B cells can also regulate the course of the disease by producing the inhibitory cytokine TGF-β (23). Moreover, Tedder et al (24). reported that splenic CD19^+^ CD5^+^ CD1d^hi^ B cells express high levels of IL-10, and adoptive transfer of sorted CD19^+^ CD5^+^ CD1d^hi^ B cells into mice with autoimmune disease reduces disease severity (24–26). Since this initial report, multiple studies have confirmed that Bregs exert negative immune regulation in diverse autoimmune settings, and that CD19^+^ CD5^+^ CD1d^hi^ B cells often exhibit quantitative and/or functional defects in autoimmune diseases (27, 28).

In humans, Bregs are more appropriately considered a functional regulatory state than a single lineage-defined subset. Nevertheless, reported human Breg phenotypes can be broadly related to canonical B cell compartments. CD19^+^CD24^hi^CD38^+^B cells are generally linked to transitional/immature or naïve-like B cells (29), whereas CD19^+^CD24^hi^CD27^+^B cells are more closely associated with memory B cell populations (29, 30). In addition, regulatory functions have also been reported in plasmablasts and plasma cells, indicating that immunoregulatory activity may coexist with antibody-secreting differentiation (31). By contrast, age-associated B cells (ABCs) are more commonly associated with inflammatory activation and should be distinguished from bona fide Breg programs (32). These findings support the concept that Bregs represent context-dependent regulatory programs that can arise across multiple canonical B cell subsets.

The dominant effector functions of Breg-like cells may differ across canonical B cell subsets. Transitional and memory-associated Bregs are most commonly characterized by anti-inflammatory cytokine secretion and their capacity to suppress pathogenic T-cell responses, promote Treg induction, and modulate antigen-presenting cell function (33). In contrast, regulatory plasmablasts and plasma cells may combine cytokine-mediated immunosuppression with the classical effector properties of antibody-secreting B-lineage cells. This coexistence of regulatory activity and antibody production highlights the functional plasticity of B cells in immune homeostasis and autoimmune disease (34) (**Table 1**).

**Table 1:** Relationship between reported human Breg phenotypes and human B cell subsets.

Human B cell subsets	Representative phenotype	Regulatory functions	Antibody production	Reference	
Immature B cells	CD19^+^CD24^hi^CD38^hi^	IL-10 production, suppression of CD4^+^ T-cell responses, promotion of Treg function	Limited	(29, 34)	
Naive B cells	inducible IL-10^+^ states under stimulation	cytokine secretion, APC modulation	generally low	(36, 37)	
Memory B cells	CD19^+^CD24^hi^CD27^+^, CD27^+^regulatory populations	IL-10 production, inhibition of Th1/Th17 responses,	Differentiate into antibody-secreting cells	(30, 38)	
Age-associated B cells	inflammatory activated states; relationship to Bregs unclear	pro-inflammatory;	Contribute to pathogenic humoral responses	(32, 39)	
Plasmablasts	IL-10-producing plasmablast-like cells	suppression, T-cell regulation	active antibody secretion	(31, 40)
Plasma cells	regulatory plasma cells	Maintenance of immune homeostasis	strong antibody secretion	(41, 42)

Additional B cell subsets with immunomodulatory functions have been described in both mice and humans in recent years. Given pronounced species-specific differences, these subsets often vary in phenotypic markers and the inhibitory molecules they express (35). Currently known Breg subsets have been summarized, and published Breg phenotypes and effector mechanisms are presented in **Table 2**. Notably, some human Breg subtypes have not been detected in murine models, whereas certain murine Breg subsets remain incompletely characterized in humans.

**Table 2:** Currently reported Breg subsets, phenotypic characteristics, and effector mechanisms.

Species	Subsets	Phenotype	Features	Function	Location	Diseases	Ref
Mouse	T2-MZP	CD19^+^ CD21^+^ IgM	IL-10	Suppresses T-cell activation and promotes tolerance	Spleen	Arthritis	(17)
	MZB	CD19^+^ CD21^hi^ CD23^-^	IL-10	Identified as an IL-10-producing marginal zone B-cell population	Spleen	Infection	(43)
	Tim-1^+^B	CD19^+^ Tim-1^+^	IL-10	Associated with maintenance of immune tolerance	Spleen	Diabetes	(44)
	B10	CD19^+^ CD5^+^ CD1d^hi^	IL-10	Defined by robust IL-10 production and suppression of inflammatory T-cell responses	Spleen	Multiple Sclerosis	(45)
	Plasma	CD19^+^ CD138^+^ IgM^+^	IL-35	Identified as regulatory plasma cells with IL-35-dependent suppressive activity	Spleen	EAE	(46)
	Plasmablasts	CD138^+^ CD44^hi^	IL-10	IL-10-producing plasmablasts shown to suppress autoimmune inflammation	Draining lymph nodes	EAE	(31)
	CD1d^hi^ B	CD19^+^ CD1d^hi^	IL-10	Identified as an IL-10-competent B-cell subset with immunoregulatory properties	Spleen	Allergic lung inflammation	(47)
	B-1a	CD19^+^ CD5^+^ CD11b^+^	IL-10	Suppresses intestinal inflammation through IL-10	Peritoneal cavity	Inflammatory Bowel Disease	(48)
	i35-Breg	CD5^+^ CD1d^hi^ FcgIIb^hi^	IL-35	Defined by IL-35 production and suppressive function	Spleen	SLE	(49)
	Killer B	CD19^+^ Fas^+^	TGF-β	Suppresses pathogenic immune responses through Fas/FasL- and TGF-β-related mechanisms	Spleen	T1D	(50)
	PD-L1^hi^ B	CD19^+^ PD-L1^hi^	IL-10	Identified as a regulatory B-cell population with PD-L1-associated inhibitory activity	Spleen	Autoimmune disease	(51)
	CD73^+^ B	CD19^+^ CD39^+^ CD73^+^	Adenosine	Mediates immune suppression through adenosine generation	Peritoneal cavity	Colitis	(52)
	CD9^+^ B	CD19^+^ CD9^+^	IL-10	Enriched for IL-10-producing regulatory activity in allergic inflammation	Spleen	allergic asthma	(53)
	IgA^+^ B	CD19^+^ IgA^+^	IL-10, TGF-β	Combines immunoregulatory cytokine production with antibody-secreting differentiation	Tumor	Colorectal tumor	(54)
Human	B10	CD19^+^ CD24^hi^ CD27^+^	IL-10	Memory-associated Breg population defined by IL-10 competence and suppression of CD4^+^ T-cell responses	Peripheral blood	rheumatoid arthritis	(55)
	Plasmablasts	CD27^hi^ CD38	IL-10	Regulatory plasmablast-like cells with IL-10-associated suppressive activity	Peripheral blood	Graft-versus-host disease	(56)
	Immature cells	CD19^+^ CD24^hi^ CD38^hi^	IL-10	Inhibitory effects on inflammatory T-cell responses	Peripheral blood	rheumatoid arthritis	(33)
	Br1	CD73^-^ CD25^+^ CD71^+^	IL-10	Associated with immune tolerance in allergy	Peripheral blood	Allergy	(57)
	CD1d^hi^ B	CD19^+^ CD1d^hi^	IL-10	Identified as an IL-10-producing B-cell population with immunoregulatory capacity	Peripheral blood	Schistosomiasis	(58)
	Tim-1^+^ B	CD19^+^ Tim-1^+^	IL-10	Enriched for IL-10-producing Bregs and associated with immune homeostasis	Peripheral blood	Myasthenia gravis	(59)
	IgA^+^ B	CD19^+^ IgA^+^	IL-10	Antibody-secreting B-cell subset with regulatory cytokine production	Peripheral blood	Inflammatory arthritis	(37)
	Killer B	CD178^+^ IL5RA	FASL	Defined by FasL-mediated suppressive or cytotoxic regulatory activity	Peripheral blood	Latent tuberculosis	(60)
	PD-L1^+^ B	CD19^+^ PD-L1^hi^	IL-10	Regulatory B-cell phenotype associated with PD-L1-dependent inhibition	Peripheral blood	Rheumatoid arthritis	(61)
	GrB^+^ B	CD19^+^ GrB^+^	Granzyme B	Identified based on granzyme B-mediated immunoregulatory function	Peripheral blood	HIV	(62)
	CD5^+^ B	CD19^+^ CD5^+^	Granzyme B	Granzyme B-producing B-cell population with regulatory potential	Peripheral blood	SLE	(63)
	CD73^+^ B	CD19^+^ CD39^+^ CD73^+^	Adenosine	Suppresses immune responses through adenosine-related pathways	Peripheral blood	–	(64)
	CD9^+^ B	CD19^+^ CD9^+^	IL-10	IL-10-associated regulatory phenotype linked to allergic inflammation	Peripheral blood	Asthma	(65)

B10 cell, IL-10-secreting Breg; Br1, Breg type 1; Breg, regulatory B cell; CD, cluster of differentiation; GrB, granzyme B; IDO, indoleamine 2,3-dioxygenase; Ig, immunoglobulin; IL, interleukin; PD-L1, programmed cell death ligand 1; TCR, T cell receptor; TGF, transforming growth factor.

## 3 The mechanisms of Breg immune suppression

**Figure 1:**
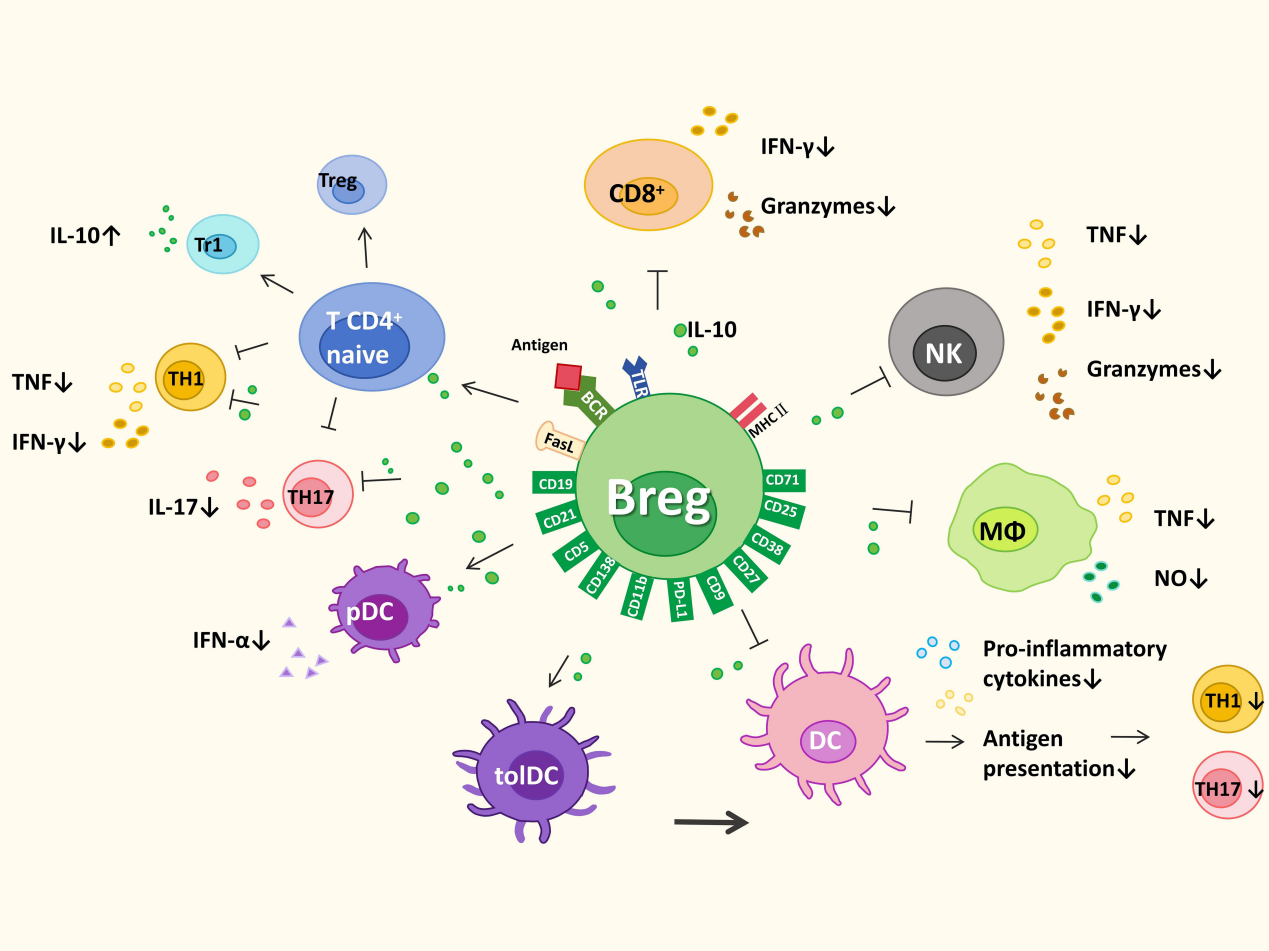
Key cytokines and inhibitory surface molecules mediating Breg immune regulation.Bregs suppress immune responses through anti-inflammatory cytokines and inhibitory surface pathways. As illustrated, IL-10, IL-35, TGF-β, PD-1/PD-L1, and Fas/FasL collectively inhibit Th1, Th17, CD8^+^ T cells, NK cells, macrophages, and dendritic cells, while promoting Treg, Tr1, and tolerogenic dendritic-cell responses. Through these mechanisms, Bregs reduce inflammatory cytokine production, restrain antigen presentation, and maintain immune tolerance.

B cells are essential for both innate and adaptive immune regulation (66). Although no single surface marker or transcription factor uniquely defines Bregs, available evidence indicates that their immunomodulatory functions are mediated primarily through secretion of anti-inflammatory factors, together with inhibitory signals delivered by surface molecules. The cytokines and surface molecules implicated in Breg-mediated immunomodulation are summarized in **Figure 1**.

### 3.1 Anti-inflammatory cytokines produced by Bregs

There has been substantial research on the inhibitory functions of Bregs. To date, IL-10, IL-35, and TGF-β are regarded as key soluble mediators of Breg immunomodulation (5).

### 3.2 IL-10

It is widely accepted that Breg function is strongly dependent on the expression and secretion of IL-10 (67). IL-10 is a potent anti-inflammatory cytokine that protects against excessive inflammatory responses to pathogens and commensal microbiota, and it also plays major regulatory roles in cancer, autoimmunity, and systemic homeostasis (68). IL-10 exerts strong anti-inflammatory effects primarily by acting on antigen-presenting cells (APCs), including monocytes and macrophages, to suppress the production of pro-inflammatory cytokines. IL-10 can also reduce APC antigen-presenting capacity by downregulating MHC-II expression and can dampen T-cell activity by inhibiting T-cell inflammatory mediator synthesis and release (69). In murine autoimmune models, Bregs suppress T-cell inflammatory cytokine production in an IL-10–dependent manner. Moreover, adoptive transfer studies support the conclusion that IL-10 can inhibit T-cell expansion, thereby functioning as a negative regulator in inflammation and autoimmune disease (70).

Kleffel (70) reported that normoglycemic NOD mice with prolonged normal glucose levels exhibited reduced lymphocytic infiltration and increased frequencies of IL-10+ B cells in pancreatic islets compared with hyperglycemic NOD mice. To define the immunomodulatory properties of IL-10^+^ B cells, splenic B cells from normoglycemic NOD mice were co-cultured with dendritic cells (DCs) and CD4^+^ T cells. IFN-γ production by CD4^+^ T cells was significantly reduced in cultures containing IL-10^+^ B cells compared with IL-10^-^ B cells. In adoptive transfer experiments, IL-10^+^ B cells co-transferred with CD4^+^ T cells into NOD recipient mice more effectively suppressed CD4^+^ T cell–mediated islet autoimmunity and maintained normoglycemia for a longer period than IL-10^-^ B cells. Similar anti-inflammatory effects of IL-10 have been reported in other autoimmune disease models, such as SLE (71) CIA (72), IBD (11) and EAE (45).

In studies of human Bregs, IL-10 has likewise been shown to inhibit T-cell proliferation and the production of inflammatory mediators such as IFN-γ and TNF (5) Notably, this inhibitory capacity is often impaired in B cells from patients with autoimmune diseases. Beyond suppression of pro-inflammatory CD4 T-cell responses, increasing evidence suggests that IL-10–producing B cells contribute to mechanisms controlling allograft tolerance in humans. In chronic graft-versus-host disease (cGVHD), patients with active cGVHD exhibit impaired B cell IL-10 production, which may relate to defective signal transducer and activator of transcription 3 and extracellular signal–regulated kinase phosphorylation. IL-10 production is not confined to a single B cell subset but is enriched within CD24^hi^ CD27^+^ and CD27^hi^ CD38^hi^ plasmablast compartments. Similar findings have been reported in allogeneic stem cell transplantation cohorts, where cGVHD patients show reduced Breg frequencies and diminished IL-10 production compared with healthy donors and those without cGVHD (73).

In addition to restraining CD4^+^ T-cell proliferation, IL-10 has been implicated in shaping T-cell differentiation. IL-10 Bregs have been reported to promote conversion of CD4^+^ T cells into immunoregulatory Tregs. In both mice (74) and human (75), increased IL-10 Bregs suppress effector T cells while enhancing Treg function, thereby contributing to immune tolerance. Although IL-10 is central to Breg-mediated regulation and has been demonstrated *in vivo* and *in vitro*, epigenetic regulation of IL-10 expression in B cells remains incompletely understood, in part due to the absence of specific Breg transcriptional markers (76).

### 3.3 IL-35

IL-35, composed of IL-12p35 and Ebi3 subunits, is a member of the IL-12 cytokine family. Its anti-inflammatory functions have been increasingly recognized. Prior studies have shown that IL-35 participates in T-cell regulation (77). Tregs can inhibit T-cell proliferation *in vitro* by controlling IL-35 expression, thereby modulating inflammatory immune responses (78). However, IL-35 produced by B cells represents a comparatively recent development in the field.

Current studies suggest that IL-35 also contributes to Breg development and function. In animal models, chimeric mice lacking expression of IL-35 subunits (p35 or EBi3) selectively in B cells develop higher EAE scores (46). Another independent study demonstrated that the IL-12p35 subunit can exert immunomodulatory effects previously attributed to IL-35. Specifically, IL-12p35 inhibited lymphocyte proliferation, induced expansion of IL-10–producing and IL-35–producing Bregs, and ameliorated autoimmune uveitis by antagonizing pathogenic Th17 responses (22). It has also been proposed that IL-35 treatment in spontaneous lupus-like disease increases Treg and Breg frequencies, elevates anti-inflammatory cytokines, and reduces pro-inflammatory cytokines (79) Together, these examples support a role for IL-35 in regulating therapeutic autoimmune inflammation (80).

IL-35 can induce lymphocyte differentiation or expansion toward IL-10–producing and IL-35–producing Bregs (22). Nonetheless, the relationship between IL-35+ Bregs and IL-10^+^ Bregs remains unresolved: it is unclear whether these represent distinct subsets or overlapping states across B cell developmental stages. Addressing these questions will be essential for advancing the study of autoimmunity (80).

### 3.4 TGF-β

The TGF-β superfamily comprises more than 35 members and plays pleiotropic roles in cell development, proliferation, differentiation, and function (81). TGF-β is widely regarded as a major immunomodulatory cytokine critical for self-tolerance and the control of autoimmunity. Innate and adaptive immune cells, including T and B cells, can produce TGF-β1. Early studies showed that TGF-β-deficient mice die from multifocal inflammatory disease (82). Subsequent work established that TGF-β inhibits differentiation of pro-inflammatory immune cells while promoting the suppressive functions of Tregs (83). Current evidence indicates that several murine and human Breg populations can also secrete TGF-β (84). Similar to IL-10 and IL-35, TGF-βis another key mediator secreted by Bregs to regulate the immune response (85). In NOD mice, Bregs induced apoptosis in Th1 cells and inhibited the activity of antigen-presenting cells by secreting TGF-β (50). Furthermore, TGF-βproduced by Bregs enhanced the expression of cytotoxic T-lymphocyte-associated antigen-4 (CTLA-4) and Forkhead box protein 3 (FOXP3) in Tregs in human Breg and Treg co-culture studies (28). This demonstrates that the regulatory function of Breg cells on Treg cells is related to TGF-β (86).

Overall, Breg-derived TGF-β appears to regulate immune responses by suppressing effector T-cell expansion and reinforcing Treg function. However, studies focusing specifically on TGF-β in B cells remain limited, and the phenotype of TGF-β–producing Bregs is incompletely characterized. With advances in cell-specific targeting of TGF-β signaling, the regulatory roles of TGF-β in immune cells are becoming clearer (87).

### 3.5 Surface-molecules expressed by Bregs

In addition to soluble mediators, Breg surface molecules contribute to inhibitory mechanisms that collectively promote and maintain immunoregulatory functions (88).

### 3.6 Tim-1

Tim-1 is a transmembrane glycoprotein and a member of the Tim family involved in immune regulation. Early studies identified Tim-1 expression in T cells and implicated it in T-cell activation and Th-cell responses (89). Recent studies have found Tim-1 expression in other immune cells, such as mast cells (90), Tregs (91), and B cells (92). Tim-1 is important for Treg development and function. Anti-Tim-1 mAb reduces FOXP3 expression, inhibits differentiation of effector T cells into Tregs, and weakens Treg-mediated immunosuppression, thereby preventing organ transplantation tolerance in mice (93).

In addition to its regulatory role on Treg cells, Tim-1 is essential for Breg function and development. In one study, researchers created Tim-1 mutant mice. Notably, in these mutant mice, the ability of Breg to produce IL-10 was impaired (94). IL-10-producing Breg cells were shown to express high levels of Tim-1 in a mouse model, and a Tim-1-specific antibody was found to induce Tim-1^+^ Breg in mice, inhibit Th1 and Th17 inflammatory factors *in vivo*, and improve over-transfer tolerance in mice (44). These findings collectively suggest that Tim-1 is required for Bregs to promote immune tolerance. In humans, Araven et al. assessed Tim-1 expression across B cell subsets and found that transitional B cells—often enriched for regulatory features—exhibit the highest Tim-1 levels, and that IL-10 production is enriched within Tim-1^+^ Bregs (95).

In humans, three Tim family members have been identified (TIM-1, -3, and -4). Tim-3 was first described on Th1 cells and functions as an inhibitory receptor that promotes Th1 cell death upon ligand engagement, thereby reducing IFN-γ production (96) Tim-4 is a natural ligand for Tim-1 and is predominantly expressed on APCs rather than T cells; it can regulate T-cell proliferation and contribute to autoimmune disease processes (97). These findings suggest that Tim-1 expressed by Bregs may interact with Tim-4 on APCs, reduce APC antigen-presenting capacity, inhibit pro-inflammatory mediator production, and promote Treg differentiation (98).

### 3.7 PD-1

PD-1 (programmed death receptor 1) is a major immunosuppressive molecule that promotes self-tolerance by downregulating immune responses and limiting T-cell inflammatory activity, thereby protecting against autoimmune disease (99). PD-1 is expressed on T cells, B cells, macrophages, and some DCs. Through binding to its ligands PD-L1 and PD-L2, PD-1 delivers inhibitory signals that regulate the balance between T-cell activation, tolerance, and immune-mediated tissue injury (100) Although PD-1 is best characterized in T cells, several studies demonstrate important functions in other cell types. Notably, PD-1 and PD-L1 are highly expressed on Tregs and contribute to their induction and suppressive functions (101) These observations have motivated exploration of PD-1/PD-L1 pathways in Breg biology (102).

In a breast cancer model, investigators identified a CD19^+^PD-L1^+^ Breg population with stronger T-cell inhibitory effects than previously described Bregs (102). In mouse colorectal tumors, IgA^+^ Bregs expressed multiple immunomodulatory molecules (PD-L1, IL-10, and TGF-β) and suppressed CD8^+^ T-cell proliferation and activation (54), suggesting a role in peripheral tolerance. In human tumor studies, PD-1–expressing B cells inhibited CD4 and CD8 T cells through a PD-1/PD-L1–dependent mechanism. This subset is rare in peripheral blood but is markedly increased in differentiated thyroid tumors. Moreover, PD-L1 expression by B cells is significantly elevated in the peripheral blood of thyroid tumor patients. *In vitro*, PD-1 B cells strongly inhibited CD4 and CD8 T-cell proliferation and viability, and this effect was reversed by PD-L1 blockade (103).

Multiple immunotherapeutic agents targeting PD-1 have been developed (104). Meanwhile, increasing evidence supports a role for PD-1 activation pathways in autoimmune disease therapy (105). PD-1 can mediate the proliferation of B cells in response to effector T-cells and IFN-γFAS: FASL-mediated apoptosis production (106). Characterization of this Breg-related pathway extends our understanding of immune regulation and provides new insights for both antitumor immunity and immunotherapy.

### 3.8 Fas/FasL

Fas and its ligand FasL are membrane-associated molecules that mediate apoptosis and have been intensively studied as regulators of activation-induced cell death during T-cell development. Fas engagement on activated T cells by FasL is a common apoptotic pathway, and Fas/FasL interactions are essential for maintaining immune homeostasis (107).

Studies in patients and animal models indicate that FAS/FASL-mediated apoptosis contributes to the pathogenesis and progression of autoimmune diseases (108). In one study, B cells expressed FasL and TGF-β following LPS stimulation, and transfer of these activated B cells into NOD mice suppressed spontaneous Th1 autoimmunity and delayed autoimmune diabetes progression (50). Another report showed that TLR4-activated Bregs inhibited CD4 T-cell proliferation *in vitro*, and this effect was blocked by anti-FasL antibody, implicating Fas-FasL signaling in Breg-mediated suppression (109). In an arthritis model, more severe arthritis correlated with reduced numbers of FasL^+^ B cells, which promote apoptosis of arthritogenic T cells without affecting protective T cells required for anti-infectious or antitumor immunity (110).

In addition, FasL^+^ B cells can induce apoptosis in non-immune cells. Anti-tumor effector B cells were shown to directly kill tumor cells via the Fas/FasL pathway (111). Collectively, these studies indicate that the Fas/FasL system is important in B cell biology (112), and impairment of this pathway in peripheral tolerance may contribute substantially to the initiation or progression of autoimmune diseases.

## 4 Molecular regulation of Breg differentiation and function

**Figure 2:**
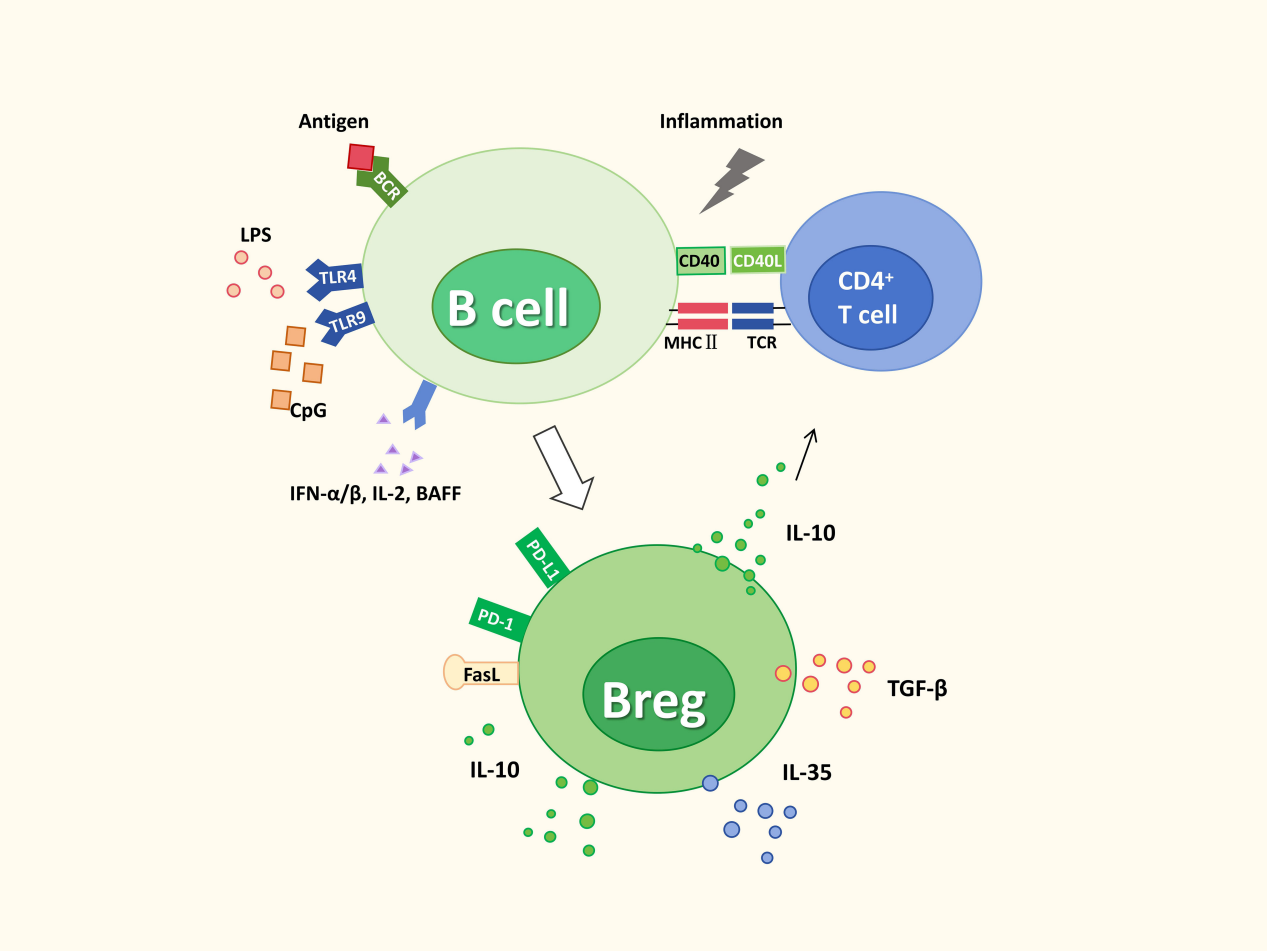
Mechanistic framework for Breg induction: extrinsic triggers, signaling integration, and regulatory mediators.Bregs arise from conventional B cells in response to combined antigenic, innate, cytokine, and T-cell-derived signals. As shown, BCR engagement, TLR stimulation by LPS or CpG, cytokines such as IFN-α/β, IL-2, and BAFF, and CD40–CD40L/MHC II–TCR interactions with CD4^+^ T cells promote Breg differentiation. Induced Bregs express inhibitory molecules, including PD-1, PD-L1, and FasL, and produce IL-10, IL-35, and TGF-β, thereby acquiring immunosuppressive function.

Under exogenous immunological stimulation, B lymphocytes can undergo functional reprogramming and differentiate into immunosuppressive Bregs. Available evidence suggests that, upon receiving helper cues from CD4^+^ T cells (e.g., CD40–CD40L interactions) together with cytokine signals within an inflammatory milieu including IL-2, IFN-α/β, and TGF-β, B cells can activate distinct transcriptional programs and acquire a regulatory phenotype. This differentiation is driven by coordinated activation of B cell costimulatory signaling inputs such as Toll-like receptors (TLRs), and is transduced primarily via downstream pathways (113). Fully activated Bregs are typically characterized by elevated IL-10, IL-35, or TGF-β and increased expression of immunoregulatory molecules including PD-1, Tim-1, and FasL, thereby suppressing effector T-cell activation, promoting Treg generation, and ultimately contributing to immune homeostasis and tolerance (**Figure 2**).

### 4.1 BCR

BCRs are antigen-specific receptors whose signals govern B cell proliferation, development, and differentiation (114). IL-10 is a key factor in negative immune regulation by B cells. Unstimulated human B cells rarely secrete IL-10, whereas BCR signaling robustly enhances IL-10 production (30). Throughout development in bone marrow and maturation in peripheral tissues, B cells undergo negative and positive selection, and BCR signaling acts as a major determinant of these processes via NF-κB and PI3K pathways (115, 116). BCR signals are also required for maintenance of immature B cells in bone marrow and mature B cells in peripheral compartments (117).

BCR signaling is also important for Breg differentiation (118). In a mouse model, Hussain et al. activated splenic B cells from NOD mice using an IgM antibody (BCR-specific stimulation) and transferred these cells into recipient NOD mice, resulting in delayed T1D onset and reduced disease incidence. Notably, infusion of BCR-stimulated B cells maintained long-term immune tolerance in NOD mice in an IL-10–dependent manner (119). In another study, CD19 transgenic mice with enhanced BCR signaling showed increased Breg numbers *in vivo*, whereas CD19-deficient mice with impaired BCR signaling generated few Bregs (24).

MHC class II, CD40, MyD88, and IL-10 receptor expression are not required for Breg development, and Bregs can develop in the absence of T cells. These findings emphasize the central role of BCR signaling in promoting IL-10 competence. One study demonstrated that BCR diversity is required for Breg development *in vivo* (120). The reduced Breg numbers observed in transgenic mouse strains with fixed BCR repertoires further suggest that appropriate antigen-specific BCR signaling is needed during Breg development. However, the strength, nature, and timing of BCR signals necessary to induce Breg differentiation and function may be tightly regulated *in vivo*. For example, BCR recognition of exogenous antigens may inhibit clonal expansion of Bregs or divert B10 progenitors toward alternative functional programs, whereas BCR signals driven by self-antigens may promote their expansion (120). Collectively, these data indicate that antigen-specific recognition via BCR is important for Breg development and function and that, depending on BCR engagement and inflammatory context, B cells may differentiate into regulatory or antibody-secreting cells to fulfill distinct biological roles (28).

### 4.2 TLR

TLR are key pattern-recognition receptors in innate immunity that link innate and adaptive responses by regulating APC activation and cytokine production (121). TLR recognize conserved microbial products and activate immune responses when pathogens breach physical barriers such as skin or mucosa (122).

TLR activation is important for B cell–derived immunoregulatory functions. Although TLR signaling can promote inflammatory responses, it also contributes to negative immune regulation, supporting immune suppression *in vivo* and maintenance of self-tolerance (123). TLR signaling enhances Breg IL-10 production by promoting B cell differentiation and expansion. In murine *in vitro* studies, TLR9 stimulation induced splenic B cells to produce high levels of IL-10 (124). Myeloid differentiation factor88 (MyD88) is a key downstream signaling molecule in TLR pathways (125), transmitting signals that influence immune regulation and disease progression (126). In one experiment, despite normal Breg development in MyD88-deficient mice, these mice failed to secrete sufficient IL-10 upon LPS stimulation (127). Studies of human peripheral blood and spleen B cells revealed that CpG-B (TLR agonist) combined with anti-Ig stimulation is a potent inducer of IL-10, and that B cells stimulated in this way inhibited CD4^+^ CD25^-^ T-cell proliferation *in vitro* partly via IL-10–dependent mechanisms (128).

Most B cells do not constitutively express IL-10, TLR agonists do not induce IL-10 in all B cells; rather, they preferentially promote IL-10 secretion by Breg-competent subsets *in vitro (*129). Taken together, TLR signaling contributes to the activation, differentiation, and function of Bregs (130).

## 5 Bregs in autoimmune diseases

In recent years, increasing evidence suggests that abnormalities in the level and function of regulatory B cells are closely associated with the development of clinical diseases. Given the central immunoregulatory role of Bregs, this review summarizes published evidence regarding Bregs in autoimmune diseases, including multiple sclerosis (MS), type 1 diabetes (T1D), rheumatoid arthritis (RA), and systemic lupus erythematosus (SLE).

### 5.1 MS

MS is the most common demyelinating disease of the central nervous system (CNS), and immune mechanisms contribute substantially to its onset and progression (131). Immune-mediated damage to myelin disrupts neural signal conduction and leads to the characteristic neurological manifestations of the disease (132). Experimental autoimmune encephalomyelitis (EAE), a widely used model of MS, first provided evidence that regulatory B cells (Bregs) can restrain CNS autoimmunity (133).

In particular, B-cell deficiency aggravates EAE and impairs recovery, supporting a protective role for B cells in limiting neuroinflammation (9) (12). These studies further showed that B-cell deficiency worsens disease because B-cell-derived IL-10 limits inflammatory cytokine responses during EAE. This suggests that b cell-derived IL-10 plays a key role in controlling autoimmunity.

Mechanistic studies further suggest that CD40-dependent signals are important for IL-10 production by B cells. B cells can be activated when CD40 on the surface of the B cell is ligated to CD40 ligand expressed on T cells. This demonstrates the role of the co-stimulatory molecule CD40 as a bridge in immune regulation between B cells and T cells (134). In addition to CD40, it has also been reported that a granulocyte-macrophage colony-stimulating factor (GM-CSF) and interleukin-15 (IL-15) ‘fusokine’ (GIFT15) can have an immunomodulatory effect on Bregs (135). Mouse splenocytes acquired immunosuppressive properties when treated with GIFT15 *in vitro*. Mice with experimental autoimmune encephalomyelitis entered into complete remission after intravenous infusion of such treated Bregs. However, this clinical remission disappeared when GIFT15 was used to treat IL-10 knockout B cells. While it is unclear whether GIFT15 induces IL-10 production in B cells or enhances the IL-10-producing B cell population, GIFT15 could serve as a direction for exploring therapies for autoimmune diseases (136). Additional experimental work has also shown that B10 cells can modulate dendritic-cell antigen-presenting capacity and suppress inflammatory T-cell cytokine production during disease initiation (45). Together, these animal studies provide mechanistic support for a regulatory role of B cells in CNS autoimmunity.

In the context of human MS, the available evidence is more consistent with the concept that impaired Breg-mediated immune regulation contributes to disease pathogenesis than with a single disease-specific Breg phenotype. Reported abnormalities include reduced frequencies and/or impaired suppressive function of IL-10-producing B-cell populations, which may weaken control of pathogenic T-cell responses and compromise maintenance of immune tolerance. These observations are broadly consistent with the mechanistic findings from EAE and support the view that defective Breg function may contribute to persistent CNS inflammation and disease progression. Overall, current evidence identifies Bregs as important regulators of CNS autoimmunity and suggests that restoration of regulatory B-cell function may represent a potentially relevant therapeutic strategy in MS (137).

### 5.2 SLE

Systemic lupus erythematosus (SLE) is a chronic systemic autoimmune disease characterized by loss of immune tolerance, multisystem involvement, recurrent flares, and abundant autoantibody production (138). Its pathogenesis is driven by profound dysregulation of both T- and B-cell responses (139). The critical role of regulatory B cells (Bregs) in SLE has been investigated in both murine models and human studies. In a lupus-prone mouse model, CD19-deficient New Zealand black/white (NZB/W) mice developed nephritic pathology earlier and exhibited significantly reduced survival compared with wild-type controls, indicating that loss of CD19+ regulatory B-cell subsets aggravates lupus-like disease (140).

Human studies likewise indicate that Breg abnormalities are closely associated with SLE disease activity. Among the reported phenotypes, CD19^+^ CD24^hi^ CD27^+^ B cells are commonly regarded as representative human Breg-associated populations. In patients with SLE, the absolute number of CD19^+^CD24^hi^CD27^+^ B cells is reduced, and the proportion of this subset is negatively correlated with the SLE Disease Activity Index (SLEDAI) (141). The same study also reported that serum IL-10 levels were elevated in active SLE and positively correlated with disease activity, whereas IL-35 levels were reduced and associated with clinical manifestations, indicating an imbalance between compensatory anti-inflammatory signaling and ineffective immune regulation. In addition, another study found that although B cells from patients with active SLE exhibited abnormally increased expression of IL-10 and TGF-β, the effective secretion of these regulatory mediators remained insufficient, suggesting a state of functional exhaustion or impaired suppressive competence of Bregs within the inflammatory microenvironment (142).

Breg dysfunction may drive disease through several interconnected immunopathogenic pathways in SLE: First, the reduction and functional impairment of Bregs weakens their ability to suppress autoreactive effector T cells. Studies have shown that Bregs are significantly decreased in SLE and are associated with clinical and immunological features; under SLE conditions, their capacity to restrain inflammatory CD4^+^ effector T-cell responses is compromised, thereby favoring persistent Th1/Th17-skewed inflammation (143). Second, defective Breg function may promote autoantibody production through the T follicular helper (Tfh)/T follicular regulatory (Tfr)–germinal center axis. Bregs influence the differentiation, distribution, and function of Tfh and Tfr cells, thereby helping maintain follicular immune homeostasis. When this regulatory restraint is weakened, Tfh-driven autoreactive B-cell clonal expansion, affinity maturation, and plasma-cell differentiation become easier to sustain, promoting high-affinity autoantibody production and immune-complex formation (144). Notably, Low-dose IL-2 therapy appears to benefit SLE mainly by restoring Treg function, improving the Tfr/Tfh balance, and reducing Th17 skewing; although Bregs are not the primary target of IL-2-based therapy, a more tolerogenic immune environment may secondarily favor Breg stability and suppressive capacity (145). However, this axis is not generally considered a dominant unifying pathway in overall SLE pathogenesis and is more appropriately interpreted as a context-dependent contributor in selected inflammatory settings. Third, an imbalance in Treg is also closely associated with the pathogenesis of SLE (144), and the synergistic interaction between Breg and Treg is involved in SLE development. Studies have shown that Bregs can promote Tregs differentiation through the production of cytokines such as IL-10 and TGF-β (146). In a mouse model of SLE complicated by atherosclerosis, a reduced proportion of Bregs was negatively correlated with an increased Th17/Treg ratio (147). Therefore, functional defects in Bregs may lead to attenuation of the anti-inflammatory cytokine axis, failure to restrain effector T-cell responses, enhanced autoantibody and immune-complex formation, and broader dysregulation of the immunoinflammatory network. Together, these mechanisms contribute to the progression of SLE.

### 5.3 RA

Breg deficiency is closely associated with RA disease activity and the extent of joint destruction, further supporting a protective role for this cell subset in disease pathophysiology. Evidence indicates that, in patients with RA, elevated serum IL-27 levels are positively correlated with disease activity and are also positively associated with plasma-cell frequency and autoantibody titers (148). As a pro-inflammatory cytokine, IL-27 has been shown to promote peripheral B cell dysfunction through activation of the mammalian target of rapamycin (mTOR) signaling pathway. Importantly, mTOR signaling is hyperactivated in B cells from RA patients, and pharmacologic inhibition of this pathway can partially restore B cell homeostasis and functional competence (148). These findings suggest that Breg dysfunction in RA may be linked to a defined inflammatory signaling axis.

*In vitro* studies provide more direct support for the protective potential of Bregs, highlighting their ability to suppress inflammatory mediator production and attenuate joint inflammation. Although Breg function is impaired in RA patients *in vivo*, functionally intact Bregs can secrete mediators such as IL-10 to inhibit the differentiation and effector functions of pro-inflammatory T-cell subsets and to modulate the activity of other immune populations. While RA-focused *in vitro* experiments detailing cytokine suppression by Bregs are not explicitly described in the cited literature, their well-established immunosuppressive roles in other autoimmune models support the reasonable inference that competent Bregs can limit the production of key arthritogenic cytokines, including TNF-α and IL-17 (149). In addition, Bregs may influence neutrophil-mediated pathology. Neutrophils extensively infiltrate RA synovial fluid and interact with macrophages to form neutrophil extracellular traps (NETs), which exacerbate synovial inflammation and joint injury by activating ATP–citrate lyase (ACLY) and nuclear factor-κB (NF-κB) signaling in fibroblast-like synoviocytes (FLSs) (150). Although direct evidence remains to be established, the broad immunoregulatory capacity of Bregs raises the possibility that they may indirectly interrupt this pro-inflammatory pathway by limiting neutrophil recruitment and/or NET formation. At the signaling level, Breg function is closely intertwined with key inflammatory pathways, including JAK/STAT and TNF signaling. Notably, acupuncture has been reported to modulate RA by targeting STAT1-mediated JAK/STAT signaling and TNF pathways, thereby exerting systemic effects across multiple immune subsets such as M1 macrophages and CD4^+^ T cells (151). Overall, available evidence suggests that restoration of Breg homeostasis and suppressive function may represent a promising adjunctive strategy for controlling inflammation and limiting structural damage in RA.

### 5.4 T1D

During the development of T1D, autoreactive B cells can undermine immune regulation and accelerate destructive immune responses by negatively regulating Tregs (152). In both patients with T1D and the NOD mouse model, regulatory B-cell abundance and IL-10-associated suppressive function are reduced, supporting the concept that Breg dysfunction contributes to breakdown of islet tolerance.

Human studies have reported significant decreases in Tim-1^+^ Bregs and IL-10^+^ Bregs in the peripheral blood of patients with T1D compared with healthy controls (153). Reductions in these Breg subsets correlated with clinical indicators of islet function: the frequencies of Tim-1^+^ Bregs and IL-10^+^ Bregs were negatively associated with fasting plasma glucose and positively associated with fasting C-peptide levels (153). In the NOD model, B cell deficiency prevents the development of T1D and can even restore pancreatic β-cell function, underscoring the central role of B cells—including both pathogenic B cell subsets and Bregs—in disease progression (154). Further work showed that NOD B cells exhibit aberrant metabolic reprogramming; despite increased glucose uptake and elevated IL-10 expression, attenuated hypoxia-inducible factor-1α (HIF-1α) signaling is accompanied by impaired immunoregulatory capacity (155). Collectively, these data indicate that quantitative and qualitative defects of Bregs are a key feature of the breakdown of immune tolerance in T1D. Consistent with this, Breg dysfunction is strongly and inversely associated with T1D progression and the severity of islet injury. Beyond numerical decline, Breg impairment is reflected in reduced suppressive capacity. In T1D, Bregs mitigate β-cell autoimmunity through IL-10 production and FOXP3-dependent pathways; genetic mutations and dysfunction of these mechanisms exacerbate autoimmunity (156).

Beyond islet tolerance per se, metabolic dysregulation can weaken Breg-mediated immunoregulation and thereby facilitate autoimmunity. In T1D, B cells exhibit abnormal glucose metabolism and metabolic stress, which can directly affect Breg function. In early obesity-associated chronic kidney disease, single-cell transcriptomic analyses revealed broad immunometabolic remodeling in the kidney, with widespread impairment of T-cell, natural killer (NK)-cell, and B cell function (157). Similarly, in pediatric T1D, the proportions of IL-10^+^CD24^hi^CD27^+^ and IL-10^+^CD24^hi^CD38^hi^ Breg subsets are significantly decreased and are negatively correlated with glycemic control indices (fasting glucose and HbA1c) (157). These quantitative deficits and functional impairments limit the ability of Bregs to restrain autoreactive T-cell activation, thereby destabilizing immune tolerance. Notably, in obesity and insulin resistance (IR), levels of CD19^+^CD24^+^CD27^+^ and CD19^+^CD24^+^CD38^+^ Bregs increase with IR severity; however, this may represent a compensatory yet dysfunctional response, as heightened activation correlates positively with IR severity and may instead contribute to the clinical progression of T2DM (158). Taken together, metabolic abnormalities can impair Breg generation, stability, and functional output, weakening their suppressive control over effector immune cells and creating permissive conditions for uncontrolled autoimmunity and the development of diseases such as T1D.

## 6 Prospects

Overall, current evidence supports the view that regulatory B cells (Bregs) represent a heterogeneous and context-dependent population that contributes to immune homeostasis through anti-inflammatory cytokines, inhibitory surface molecules, and dynamic interactions with other immune cells, including T cells and dendritic cells (158). Importantly, Breg dysfunction is unlikely to be a static abnormality; rather, it may act both as a driver and as a consequence of autoimmune pathology in a disease stage and microenvironment dependent manner (158). Despite substantial progress, several key challenges remain unresolved. First, there is still no lineage-defining marker or transcriptional program that can reliably distinguish stable Bregs from transiently induced regulatory B cell states. Second, the phenotypic and functional heterogeneity of Bregs across tissues, disease stages, and inflammatory settings remains insufficiently characterized. Third, many reported Breg subsets are defined largely by descriptive phenotypes, whereas rigorous functional validation in disease-relevant contexts is still limited. Future studies should therefore prioritize the establishment of standardized criteria for Breg identification, the integration of single-cell and spatial multi-omics approaches to resolve disease-specific regulatory states, and the elucidation of the metabolic and molecular programs that govern Breg stability, plasticity, and suppressive capacity. From a translational perspective, these efforts may facilitate the development of Breg-oriented therapeutic strategies, including ex vivo expansion of functional Bregs, targeted induction of endogenous regulatory programs, metabolic reprogramming, and engineered cell-based approaches. Collectively, a deeper mechanistic understanding of Breg heterogeneity and immunosuppressive networks may help transform Bregs from an immunological concept into a clinically actionable strategy for restoring durable immune tolerance in autoimmune disease (159).
